# Patterns of ribosomal protein expression specify normal and malignant human cells

**DOI:** 10.1186/s13059-016-1104-z

**Published:** 2016-11-24

**Authors:** Joao C. Guimaraes, Mihaela Zavolan

**Affiliations:** Computational and Systems Biology, Biozentrum, University of Basel, 4056 Basel, Switzerland

**Keywords:** Ribosomal proteins, Ribosome heterogeneity, Translation, Hematopoiesis, Cancer

## Abstract

**Background:**

Ribosomes are highly conserved molecular machines whose core composition has traditionally been regarded as invariant. However, recent studies have reported intriguing differences in the expression of some ribosomal proteins (RPs) across tissues and highly specific effects on the translation of individual mRNAs.

**Results:**

To determine whether RPs are more generally linked to cell identity, we analyze the heterogeneity of RP expression in a large set of human tissues, primary cells, and tumors. We find that about a quarter of human RPs exhibit tissue-specific expression and that primary hematopoietic cells display the most complex patterns of RP expression, likely shaped by context-restricted transcriptional regulators. Strikingly, we uncover patterns of dysregulated expression of individual RPs across cancer types that arise through copy number variations and are predictive for disease progression.

**Conclusions:**

Our study reveals an unanticipated plasticity of RP expression across normal and malignant human cell types and provides a foundation for future characterization of cellular behaviors that are orchestrated by specific RPs.

**Electronic supplementary material:**

The online version of this article (doi:10.1186/s13059-016-1104-z) contains supplementary material, which is available to authorized users.

## Background

Protein synthesis is at the core of cellular life. It is carried out by the ribosome, a highly conserved molecular machine with the same basic architecture in all free-living organisms [1–3]. In humans, the ribosome is composed of four ribosomal RNAs (rRNAs) and 80 ribosomal proteins (RPs), and its structure is believed to be largely invariant [4]. However, recent studies have started to uncover some degree of variability in ribosomal components, such as at the level of rRNA modifications and RP expression. These have also been linked to both ribosomal function and the physiological state of cells (reviewed in [5, 6]).

Variability in ribosomal components could lead to a vast number of ribosome variants. Alternatively, the different components may have extra-ribosomal functions, as some ribosomal proteins do. Since synthesis of translational machinery components represents a large part of the energetic cost of cellular life, the abundance of ribosomal proteins is expected to be under tight control. Indeed, many feedback mechanisms have been discovered that link the production of different ribosomal components to maintain an appropriate stoichiometry [7]; a number of RPs act in negative feedback loops to control their own expression as well as the expression of other RPs, either at the level of splicing [8, 9] or at the level of mRNA decay [10]. In bacteria, RPs frequently regulate, in negative feedback, the translation of entire RP operons [11, 12]. In eukaryotes, imbalanced RP levels frequently engage the p53 pathway [13–15] to cause cell cycle arrest and apoptosis. Aside from the feedback on ribosome biogenesis, perturbed expression of distinct RPs elicits a broad spectrum of phenotypes, from developmental defects to diseases [5, 6].

Interestingly, analysis of mRNA abundance revealed considerable differences in RP expression across human tissues [16], in mouse development [17], and in cancers [18–24]. However, the functional significance of such variation, as well as the underlying RP-dependent regulatory mechanisms, has remained insufficiently studied. Insights into the physiological roles of specific RPs have mostly come from naturally occurring phenotypes or diseases associated with RP loss of function. For instance, Rpl38, a component of the large ribosomal subunit, is essential for the appropriate axial skeleton formation in mouse embryonic development [17]. The 5′ untranslated regions (5′UTRs) of *Hox* mRNAs contain sequence elements that prevent translation of the corresponding transcripts unless the Rpl38 protein enables the recognition of their internal ribosome entry site (IRES)-like elements [25]. Furthermore, a striking number of human hematological disorders such as Diamond-Blackfan anemia (DBA) [26], T-cell acute lymphoblastic leukemia [27], and the 5q- syndrome [28] have been linked to mutations or chromosomal deletions which cause RP deficiencies. The consequences can be remarkably circumscribed, as in the case of RPS19, an RP frequently mutated in DBA patients, whose haploinsufficiency leads to reduced translation of *GATA1* mRNA [29] and subsequent defects in erythrocyte maturation.

Cancer cells have a remarkable ability to evade anti-tumorigenic signals that control normal tissue architecture and progress into a chronic proliferation program [30]. The high demand for protein synthesis in rapidly dividing malignant cells leads to increased ribosome biogenesis [31]. Surprisingly, however, dysregulation of specific RPs has been observed in both cancer cell lines and patient samples [18–24]. The roles of individual RPs seem rather difficult to predict. For example, by binding to the 5′UTR of *p53* mRNA and enhancing its translation, RPL26 can trigger programmed cell death [21]. The ectopically overexpressed RPL36A has an entirely different function, localizing to nucleoli and increasing colony formation and cell growth of hepatocellular carcinoma lines, presumably through a more rapid cell cycling program [20]. Thus, some RPs can act as tumor suppressors, whereas others promote tumorigenesis.

Despite the increasing body of evidence that individual RPs have cell-type-specific functions, a comprehensive study of RP expression heterogeneity across human cells has not been carried out so far. Furthermore, the factors that drive differential RP expression in distinct cellular contexts remain largely unknown. Through a comprehensive analysis of human RP expression pattern across 28 tissues, more than 300 primary cells, and 16 tumor types, we here estimate that about a quarter of RP genes exhibit tissue-specific expression. We find a particularly high RP expression heterogeneity in the hematopoietic system, where a small number of RP genes unequivocally discriminate cells of distinct lineages and developmental stages. Our analysis of transcription regulatory elements located in the promoters of RP genes indicates that key hematopoietic transcription factors could orchestrate the observed patterns of RP expression. Strikingly, we uncover a consistent dysregulated expression of individual RPs across cancers, which can be partially explained by copy number alterations. Our analysis suggests prominent roles of specific RPs in health and disease.

## Results

### RP genes are differentially expressed across tissues

**Fig. 1:**
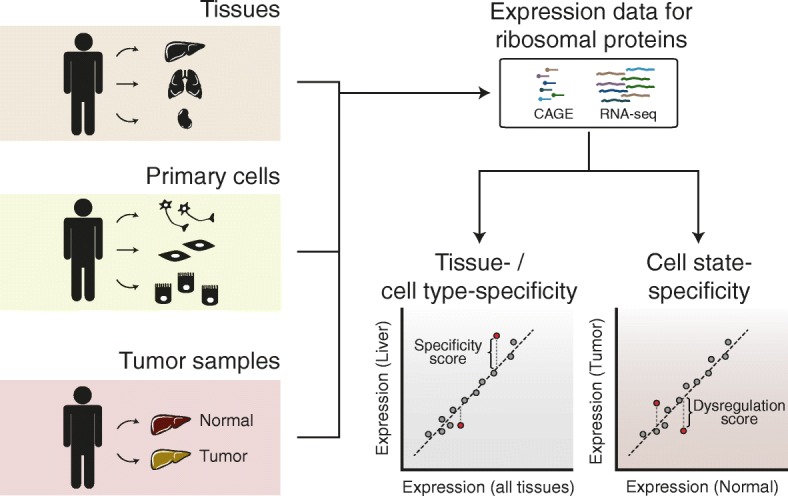
Evaluation of RP expression specificity across human tissues, primary cells, and tumors. The expression levels of RP genes across human tissues and primary cells were obtained from the FANTOM5 project repository. Gene expression data for matched normal and tumor samples were retrieved from The Cancer Genome Atlas (*TCGA*). As the correlation of RP expression levels across samples was very high, tissue/cell-specific expression of individual RP genes was estimated relative to the average across samples. Similarly, the scores for RP dysregulation in tumors were computed relative to the linear fit to the RP expression in the matched normal tissue. Cap analysis gene expression (*CAGE*) is based on the purification and sequencing of the 5′ end of mRNAs

We used the promoter-level expression atlas generated by the FANTOM Consortium [32] to evaluate the expression of 90 distinct RP genes, including 19 paralogs, across adult human tissues (Fig. 1). Although the mRNA levels of RPs within a tissue spanned a wide range, two (brain) to three (blood) orders of magnitude (Additional file 1: Figure S1a), they were highly correlated between tissues (median Pearson correlation coefficient *R* = 0.96), consistent with a strongly conserved stoichiometry of ribosomal components across tissues. The total RP expression was strongly correlated with the proliferation index of the tissue (*R* = 0.81, *P* < 0.0001, Additional file 1: Figure S1b), indicating that the proliferative state of cells explains most of the variability in global RP expression across tissues.

**Fig. 2:**
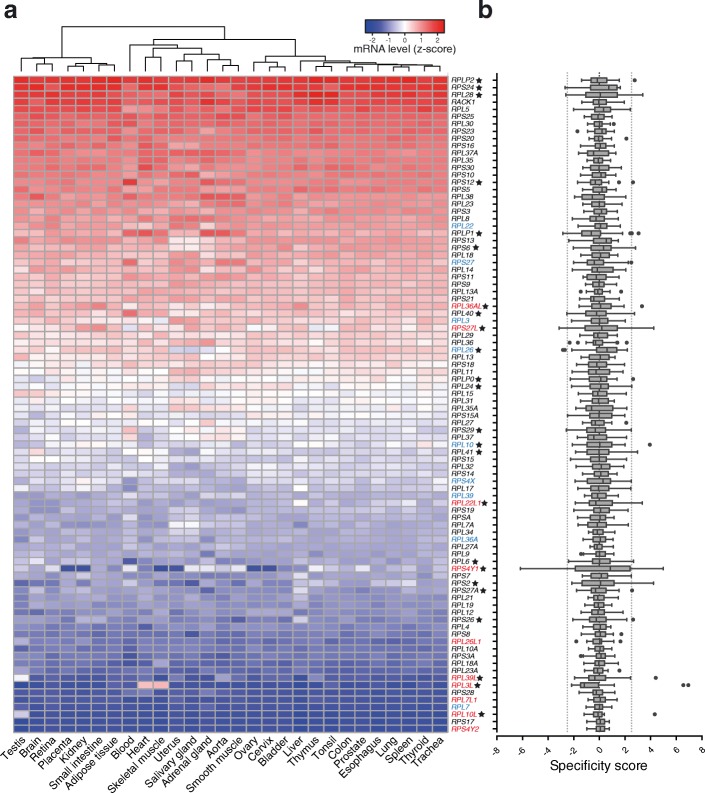
Several RPs exhibit tissue-specific expression. **a** Heatmap depicting the standard score-normalized expression level of 90 RP genes across 28 human tissues. Genes encoding the same RP (paralog genes) can be classified into canonical (e.g., because they are more ubiquitously expressed) and non-canonical and are highlighted in *blue* and *red*, respectively. RP genes exhibiting tissue-specific expression are marked with a *star*. **b** Boxplots illustrating the distribution of specificity scores for each RP across tissues. Boxes extend from the 25^th^ to 75^th^ percentiles (interquartile range (*IQR*)), *vertical lines* represent the median, *whiskers* indicate the lowest and highest datum within 1.5*IQR from the lower and upper quartiles, respectively. Values outside this range are plotted as individual *dots. Dotted lines* show the thresholds defining tissue-specific RPs (|specificity score| >2.5). RPs are shown in the same order as in panel **a**

**Fig. 3:**
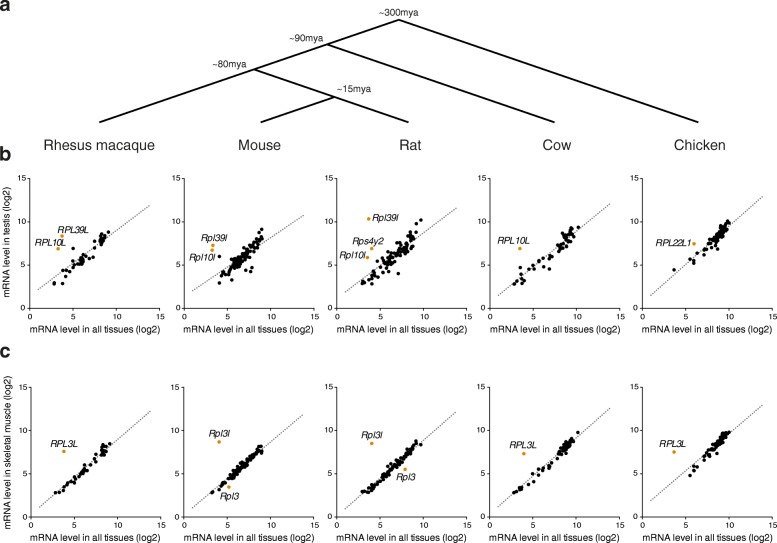
Differential expression of RPs is conserved across evolutionarily distant vertebrates. **a** RP expression was examined for five different vertebrates (rhesus macaque, mouse, rat, cow, and chicken) that have diverged ~300 million years ago. **b**, **c** RP mRNA expression levels in testis (**b**) and skeletal muscle (**c**) compared to the average expression levels across all tissues in five different vertebrates. Each *dot* is an RP gene, and the linear fit is shown as a *dotted line*. RPs displaying significant tissue-specific expression are shown in *orange* and labeled. Increased expression of *RPL10* and *RPL39L* in testis is conserved in human, rhesus macaque, mouse, and rat. *RPL10L*, but not *RPL39L*, is also expressed at higher level in cow. In chicken, another non-canonical RP gene paralog, *RPL22L1*, is specifically expressed in testis. Skeletal muscle samples exhibit a striking up-regulation of the non-canonical RP gene paralog *RPL3L* across all five species

Once the global tissue-dependent effect on expression levels was removed, the standard score-normalized expression of each RP was very consistent across tissues, with some notable exceptions (Fig. 2a). Tissue-specific patterns of RP expression are also evident at the protein level (Additional file 1: Figure S1c–f). For example, the expression of *RPL3L*, a paralog of the canonical ribosomal protein *RPL3*, is markedly higher in skeletal muscle — where it was found to regulate growth [33] — compared to other tissues. Similarly, the testis-specific RPL39L has been shown to be present in the ribosomes isolated from testis but not from other rodent tissues [34]. The conservation of these expression patterns across different vertebrates further attests their physiological relevance (Fig. 3).

To systematically evaluate the tissue specificity of RP expression, we computed ”specificity scores,” defined as the deviation of RP expression levels in each particular tissue from the average across tissues (Fig. 1). Positive and negative scores indicate a higher and lower than expected expression level in a specific tissue, respectively. At a specificity score threshold corresponding to 2.5 standard deviations (SDs), 24 of the 90 (~27%) human RP genes exhibit tissue specificity of expression (Fig. 2b, Additional file 2: Table S1). Importantly, specificity scores for the different human tissues can be reproduced using a different tissue expression atlas (Additional file 1: Figure S1g). We identified both relatively up- (~66%) and down-regulated (~33%) RP genes. Interestingly, paralogs were particularly enriched among RP genes with tissue-dependent expression (*P* < 0.05, Fisher’s exact test), as may be expected if they underwent functional specialization. RP paralogs are highly similar but generally not identical to their canonical counterparts (Additional file 1: Figure S1h). Although only about a quarter of all RPs showed evidence of tissue-specific expression in human, for about half of the investigated tissues the specificity scores of all RPs were significantly correlated between different vertebrates (Additional file 1: Figure S2).

### Extensive RP expression heterogeneity in the hematopoietic system

**Fig. 4:**
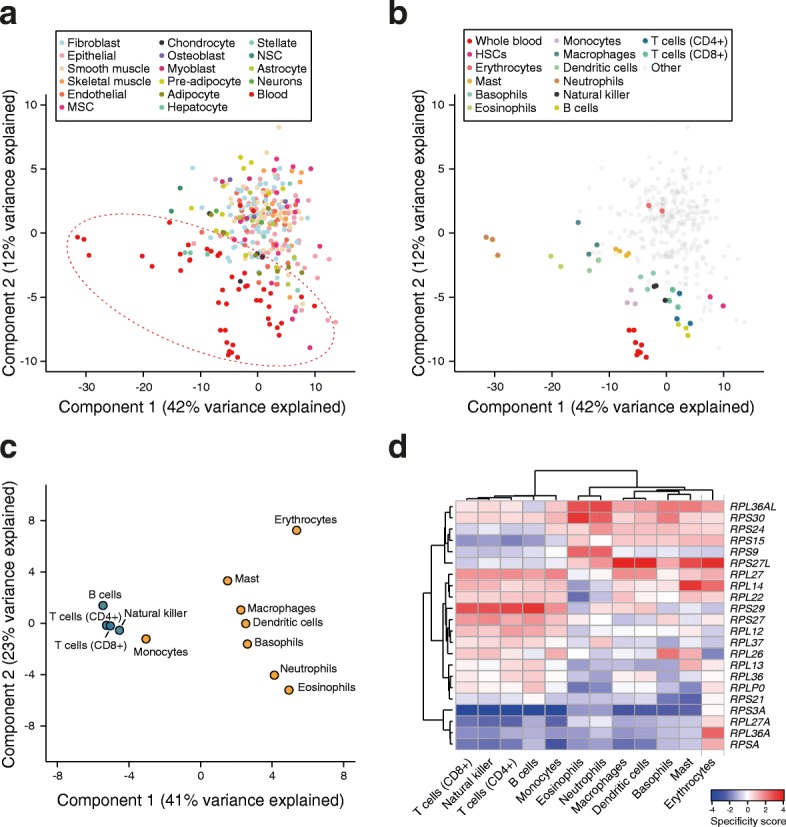
The pattern of RP expression distinguishes hematopoietic cell types. **a** PCA reveals distinct RP expression patterns in primary hematopoietic cell samples (*red dashed oval*) and samples from other cell types (*color code in the legend*). *MSC* mesenchymal stem cell, *NSC* neural stem cell. **b** Hematopoietic cell types have been recolored to illustrate that the pattern of RP expression can discriminate cells belonging to different developmental states. *HSC* hematopoietic stem cell. **c** PCA of RP specificity scores of mature cell types compared to HSCs identifies hematopoietic lineages (*blue*: lymphoid cell types, *orange*: myeloid cell types). **d** Hierarchical clustering of specificity scores across cell types suggests that several RP genes are co-regulated in the different developmental lineages

Profiling gene expression at the level of tissues, which are complex environments populated by a myriad of cell types, likely obscures differences between distinct cell types. Therefore, we have further analyzed expression of RP genes in more than 300 samples from human primary cells originating from all the three germ layers [32]. Principal component analysis (PCA) revealed two main clusters (Fig. 4a), one very broad and almost entirely composed of hematopoietic cell types, and the other more compact, containing all remaining cell types. Distinct primary hematopoietic cells were also clearly distinguishable in the PCA (Fig. 4b): lymphoid cell types (natural killer, T, and B cells) formed a compact cluster, whereas myeloid cell types were more scattered. Samples from hematopoietic precursors also clustered together and away from differentiated cells. Finally, pooled blood samples were highly similar and clearly distinguishable from individual cell types, underscoring the importance of analyzing primary cells to grasp the degree of RP expression heterogeneity across elementary cell types.

Aggregating hematopoietic samples by cell type and performing PCA of the specificity scores of each RP in each cell type relative to hematopoietic stem cells revealed two distinct clusters corresponding to the lymphoid and myeloid lineages (Fig. 4c). Hierarchical clustering showed that multiple RPs appear to be coordinately regulated in subgroups of related cell types (Fig. 4d). For instance, *RPS29* and *RPS27L* exhibit antagonistic expression patterns in lymphoid and myeloid cell types, whereas *RPL36A* and *RPS3A* are more specific for mature erythrocytes. Interestingly, precisely the opposite pattern is observed when comparing *RPS29* and *RPS27L* expression in cell-line models of lymphoma/lymphoid leukemia, which represent intermediate developmental stages, and mature lymphocytes (Additional file 1: Figure S3). This strengthens the hypothesis of a connection between RP expression regulation and the hematopoietic developmental program. Similarly, RP genes with myeloid-lineage specificity in our analysis, such as *RPS27L* [35], *RPS15* [36], and *RPS24* [37], have been previously implicated in bone marrow deficiencies.

### Promoters of hematopoietic lineage-specific RPs display distinct regulatory signatures

The intriguing observation that RPs exhibit tissue-specific and cell-type-specific variations in expression raises the question of what transcriptional regulators are responsible for these changes. Recent work revealed the complexity of transcriptional regulation of human genes, most of which have multiple promoters that are selectively activated in a tissue-specific manner [32]. The promoter-level expression atlas allowed us to examine the usage of individual RP gene promoters (1 to 16 per RP, with a median of 3) across tissues and primary hematopoietic cells. Surprisingly, although cases of single and multiple active promoters for a given RP are represented in the data (Additional file 1: Figure S4a,b,c), the tissue and hematopoietic cell-type-specific RP genes do not show an increased usage of alternative promoters with respect to non-specific RPs (Additional file 1: Figure S4d,e,f).

**Fig. 5:**
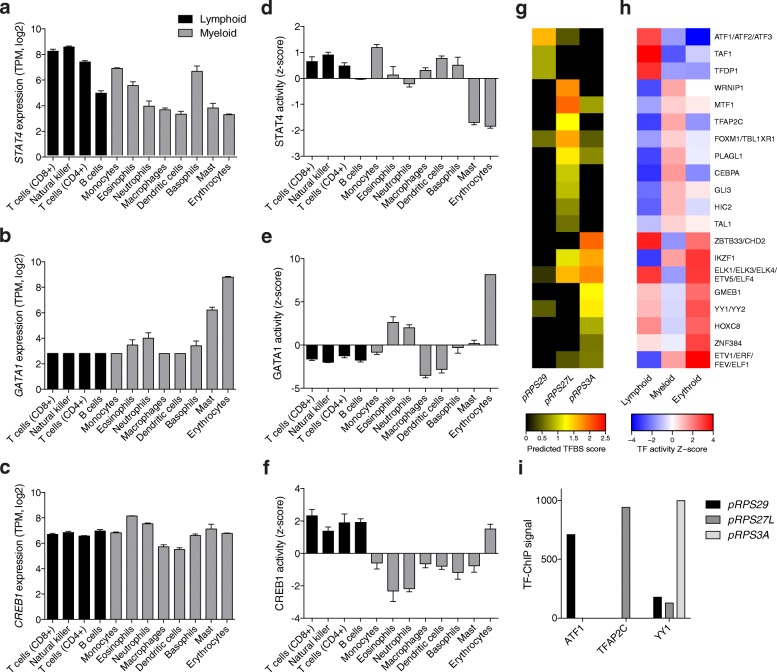
Differentially expressed RPs display distinct regulatory fingerprints. **a–c** Expression level of *STAT4* (**a**), *GATA1* (**b**), and *CREB1* (**c**) across the different hematopoietic cell types. Lymphoid and myeloid cell types are colored in *black* and *gray*, respectively. *STAT4* (*P* < 0.001) and *GATA1* (*P* < 0.01) show an expression bias toward lymphoid and erythroid cells, respectively. Conversely, the expression of *CREB1* is not significantly different between lymphoid and myeloid cell types (*P* = 0.55). **d**–**f** Differences in the activities of STAT4 (**d**), GATA1 (**e**), and CREB1 (**f**) across hematopoietic cell types, as inferred from the regulation of their respective targets. STAT4 and CREB1 show a higher activity in lymphoid compared to other cells (*P* = 0.09 and *P* < 0.001, respectively), whereas GATA1 shows a greater activity in erythrocytes (*P* < 0.01). The distributions of expression levels and activities of transcription factors between cell types of different lineages were compared using the non-parametric Mann-Whitney U test (one-tailed). **g** The heatmap of the predicted transcription factor binding site (*TFBS*) motif scores (*rows*) in the promoters of the three RP genes (*columns*) that had the highest variance across the different primary hematopoietic cells indicates largely non-overlapping transcription regulatory interactions. **h** Heatmap of the average activity scores across cell types belonging to the three different lineages (lymphoid, myeloid, and erythroid) for the TFs listed. **i** TF-ChIP-inferred binding scores of three different lineage-specific TFs (ATF1, TFAP2C, and YY1 for lymphoid, myeloid, and erythroid lineages, respectively) in the promoters of three different RPs displaying lineage-specific expression (*RPS29*, *RPS27L*, and *RPS3A* for lymphoid, myeloid, and erythroid lineages, respectively)

We next examined the predicted binding sites of 495 transcription factors (TFs) [38] and found a surprising degree of heterogeneity in the number of RP promoters predicted to be targeted by individual TFs (Additional file 1: Figure S5a). This suggests that TFs may provide context-specific regulation of RP genes. To test this hypothesis, we first determined the TFs that are most specifically expressed in the different hematopoietic lineages. As expected, previously described lineage-specific TFs showed a strong expression bias toward the corresponding cell types: STAT4 for the lymphoid [39] (Fig. 5a) and GATA1 for the erythroid [40] (Fig. 5b) lineages. Other well-known cell-type-specific TFs such as CREB1, which is active in lymphoid cells [41, 42], did not show particularly strong expression bias (Fig. 5c), possibly because it is its activity, rather than its expression level, that changes between cell types. Indeed, if we estimate the activity of TFs by modeling the expression level of their targets [38], we can identify TFs whose activity changes in a cell-type-specific manner (Fig. 5d–f), even when the corresponding expression levels remain invariant (Fig. 5c and f).

We then asked whether binding motifs of TFs with lineage-specific activities are enriched in the promoters of RP genes that exhibit a matching lineage specificity of expression. Remarkably, as shown for three RP genes with the largest variance in specificity scores across developmental lineages: the lymphoid-specific *RPS29*, myeloid-specific *RPS27L*, and erythrocyte-specific *RPS3A* (Fig. 4d), their promoters are predicted to be preferentially targeted by TFs with a matching lineage-specific activity (Fig. 5g,h and Additional file 1: Figure S5b). Although data pertaining to the direct interaction of TFs with RP promoters in individual hematopoietic lineages are lacking, chromatin immunoprecipitation studies carried out by the Encyclopedia of DNA Elements (ENCODE) consortium [43] confirm that the TF-RP promoter interactions that we predicted here do occur at least in some cell lines in which these TFs are expressed (Fig. 5i). Generalizing the analysis to comprehensive sets of RPs that exhibit lymphoid, myeloid, or erythroid specificity of expression leads to a similar result (Additional file 1: Figure S5c–n). Altogether, our analysis indicates that cell-type-specific patterns of RP expression could be explained by the binding of multiple TFs with lineage-restricted activity to individual RP promoters.

### Dysregulated expression of specific RP genes in cancers

**Fig. 6:**
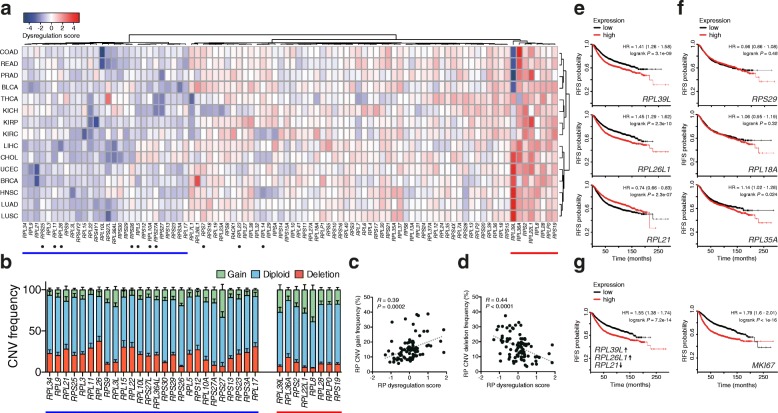
Dysregulation of RP expression in cancers is linked to survival. **a** Heatmap showing the dysregulation scores of individual RP genes in cancers. There are three prominent clusters of RPs, corresponding (*from left to right*) to consistently negative (*blue line*), variable, and consistently positive (*red line*) dysregulation score across different cancers. RPs reported to be involved in p53 regulation are marked with a *dot*. Several cancers also exhibit dysregulation of specific RP genes. *COAD* colon adenocarcinoma, *READ* rectum adenocarcinoma, *PRAD* prostate adenocarcinoma, *BLCA* bladder urothelial carcinoma, *THCA* thyroid carcinoma, *KICH* kidney chromophobe, *KIRP* kidney renal papillary cell carcinoma, *KIRC* kidney renal clear cell carcinoma, *LIHC* liver hepatocellular carcinoma, *CHOL* cholangiocarcinoma, *UCEC*, uterine corpus endometrial carcinoma, *BRCA* breast invasive carcinoma, *HNSC* head and neck squamous cell carcinoma, *LUAD* lung adenocarcinoma, *LUSC* lung squamous cell carcinoma. **b** RP genes showing negative (*blue line*) and positive (*red line*) dysregulation score exhibit a correspondingly high degree of copy number variation (*CNV*) deletion and gain across cancers, respectively. Error bars depict the standard error of the mean. **c**, **d** RP average dysregulation scores across cancers are significantly correlated with both the average frequency of copy number gain (**c**) and deletion (**d**). **e**, **f** Kaplan-Meier relapse-free survival (*RFS*) plots for three RPs identified as dysregulated (**e**) or not dysregulated (**f**) in human breast carcinoma. **g** Kaplan-Meier RFS plots for the combined signature of the three RPs identified as dysregulated in breast cancer (*left*) and the gene that is most predictive for breast cancer RFS: *MKI67* (*right*). Hazard ratios (*HR*) and respective 95% confidence interval as well as logrank *P* values are shown for each survival analysis

We next used the The Cancer Genome Atlas (TCGA) data to compare RP expression levels in matched normal and malignant tissue samples. Notably, we observed a median increase of ~30% in the median RP expression levels of tumors as compared to the corresponding normal tissue samples (Additional file 1: Figure S6a). Some cancers, such as bladder and breast carcinomas, did not exhibit an overall increase in RP expression, a possible reason being that the cancer induces alterations into the adjacent tissue, which still looks ”normal” at the histological level. We then estimated dysregulation scores for each individual RP by comparing the expression levels in matched normal and tumor tissues and found an intriguing consistency across cancers (Fig. 6a). The variation in RP expression could be partially explained by copy number variation (Fig. 6b–d), indicating that the dysregulation of RP genes in cancer is strongly driven by genomic alterations. Several RP genes, some of which had been previously associated with p53 activation [6], consistently exhibited negative dysregulation scores across cancers (Fig. 6a, blue line). These RPs probably act as tumor suppressors. In the gene cluster exhibiting a consistent positive dysregulation score in distinct cancer types (Fig. 6a, red line) we identified proteins that have been previously associated with increased proliferation phenotypes, such as RPL36A [20] and RPS2 [44]. Remarkably, the average RP dysregulation scores estimated across cancers were significantly anti-correlated with the enrichment of the corresponding single guide RNA (sgRNA) in a CRISPR screening for cell viability [45] carried out in a melanoma cell line (Additional file 1: Figure S6b). This provides independent evidence for the role of specific RPs in the viability of cancer cells. *RPL39L*, an RP gene paralog with testis-specific expression (Fig. 2a), which was previously found up-regulated in hepatocellular carcinoma and in some cancer cell lines [24, 46], had the most striking bimodal pattern of expression across carcinoma types. *RPL39L* is also consistently up-regulated in several cell-line models of breast and lung carcinoma, indicating that these cell lines could be used to study the function of this protein in tumorigenesis (Additional file 1: Figure S6c).

Our systematic analysis further revealed that numerous RPs exhibited strong dysregulation only in particular cancer types (Fig. 6a). Among these, *RPL26L1* and *RPS27L* were exclusively up-regulated in breast and thyroid carcinomas, respectively, whereas *RPL21* had decreased expression in breast and uterine cancers. Strikingly, some RP gene knockouts, including *RPL21*, have been positively selected in a CRISPR-based viability screen carried out in a melanoma cancer cell line (Additional file 1: Figure S6b) [45]. This indicates that RP gene loss is not always detrimental for cellular fitness. To further evaluate the relevance of RP expression dysregulation, we examined the relationship between patients’ relapse-free survival and the expression levels of the RP genes that we inferred to be relevant for breast carcinoma. We found that high levels of *RPL39L* and *RPL26L1* and a low level of *RPL21* are associated with significantly lower patient relapse-free survival rates (Fig. 6e), whereas the expression levels of other RPs, not found to be dysregulated in patients, showed a much milder or insignificant association with prognosis (Fig. 6f). A classifier combining the expression signature of *RPL39L*, *RPL26L1*, and *RPL21* has an even greater predictive power, comparable to that of genes that are most predictive [47] for the relapse-free survival of breast cancer patients (Fig. 6g). Our data highlight the potential of using RP expression signatures for predicting disease progression.

## Discussion

In view of the strong selection pressure for translation accuracy, the ribosome has largely been viewed as an invariant, highly optimized molecular machine. Its components exhibit relatively little expression variability in populations of identical cells [48]. Therefore, the finding that a myriad of phenotypes can emerge from modulating expression of ribosomal components in *Saccharomyces cerevisiae* came as a surprise [49]. In support of the functional specialization of different RPs, gene expression analyses have reported the differential expression of RPs at the organ level both in humans and mice [16, 17]. Additionally, a few studies have started to reveal an association between individual RPs and tumorigenic phenotypes [18–24]. However, the full extent of RP expression heterogeneity can only be appreciated by systematically analyzing individual cell types and distinct biological contexts. Here we undertook this characterization in a large set of human tissues, primary cells, and cancer samples (Fig. 1).

As hinted in previous reports [16, 50], we found that human tissues display a notable level of RP expression heterogeneity (Fig. 2), with around one quarter of all RP genes exhibiting tissue-specific expression. Attesting for their functional relevance, these expression patterns are also conserved between vertebrate species that are hundreds of million years apart (Fig. 3). As may be expected, RPs exhibiting tissue-specific expression tend to be paralogs of canonical RPs, to which they are highly similar in sequence. Although it is tempting to speculate that these duplicated genes were co-opted for extra-ribosomal functions in the tissues in which they are expressed, at least in some cases these RP paralogs are found in ribosomes [34], strongly suggesting that they actively engage in translating complexes. An intriguing possibility is that up-regulated non-canonical RP gene paralogs compete with their canonical counterparts during ribosome biogenesis to yield ”specialized ribosomes” that enable selective translation of mRNAs [5, 51].

Profiling gene expression in complex tissues/organs has likely masked differences between individual cell types. Indeed, analyzing the data from more than 300 human primary cells (Fig. 4), we found a high degree of RP expression heterogeneity in hematopoietic cells, where a small subset of RPs can discriminate cell types belonging to different hematopoietic lineages. Consistently with our results, ribosomopathies, which are disorders resulting from defective ribosome function, often lead to bone marrow failure phenotypes (reviewed in [52]). In remarkable agreement with our predictions, the depletion of some of the RPs found here to exhibit lineage-biased expression, such as *RPS15* [36], *RPS24* [37], and *RPS27L* [35], has been associated with malignancy and abnormalities in the maturation process of the respective cell types. Nonetheless, some of the RPs whose disruption was previously linked to erythroid differentiation defects, such as RPS19 in DBA [26] and RPS14 in 5q- syndrome [28], did not display any noticeable expression specificity in our analysis, at least in the mature cell types analyzed. The reason for this discrepancy is unclear, one possibility being that these proteins exert their specific functions at stages of erythropoietic development that were not captured in the promoter atlas. Nevertheless, our analysis strongly suggests that some RPs are involved in the specification of hematopoietic lineages through mechanisms that largely remain to be uncovered. A first hint was offered by a recent study which showed that haploinsufficiency of RPS19 leads to specific translation defects in the *GATA1* mRNA [29], a master transcriptional regulator of erythropoiesis. Understanding the basis of this strong translation selectivity remains a challenge. Crosslinking and immunoprecipitation [53] of individual RPs may enable the discovery of their specific targets and the inference of interaction determinants.

An outstanding question still is what upstream regulators modulate the expression of RPs in different cell types. We found little evidence for differential promoter utilization in the modulation of RP gene expression across different tissues and cell types. Rather, we observed that the promoters of RPs that are differentially expressed in primary hematopoietic cells display unique regulatory fingerprints (Fig. 5). Interestingly, a recent study has shown that GATA1 TF binds to the promoter of *RPS19* in primary human erythroid cells [54], which in turn has been shown to be essential for the efficient translation of the *GATA1* mRNA [29]. Our results thus suggest that key transcriptional regulators orchestrate the production of cell-type-specific transcripts, including those encoding ribosomal proteins.

We observed intriguing patterns of RP expression in cancers. Several RP genes, some of which had been previously associated with p53 activation, consistently exhibited negative dysregulation scores across cancers (Fig. 6). These RPs may thereby act, directly or indirectly, as tumor suppressors. In some cases, the underlying molecular mechanisms have been described. For example, RPL5 [13] and RPL11 [14] have the capacity to translocate to the nucleoplasm, where they bind to H/MDM2, preventing p53 ubiquitination and degradation, whereas RPL26 enhances the translation efficiency of the *p53* mRNA [21]. In the cluster of RPs that have positive dysregulation scores across distinct cancers we identified several proteins, some of which had been previously associated with increased proliferation phenotypes, such as RPL36A [20] and RPS2 [44]. Surprisingly, we found that *RPL39L*, an RP gene paralog exhibiting testis-specific expression (Fig. 2), is consistently up-regulated in several carcinomas. The function of RPL39L, which differs from its paralog by only four amino acids, remains to be characterized. Although it is not entirely clear why increased expression of these particular RPs would confer a selective advantage to malignant cells, one could speculate that they are directly involved in selective activation of oncogenes [55] or inhibition of tumor suppressors [56]. Most unexpected was that a number of RPs exhibited strong dysregulation only in particular cancers, which suggests idiosyncratic responses of RPs to specific cellular microenvironments. For instance, although RPL26 was reported to bind the 5′UTR of *p53* mRNA to enhance its translation in a breast cancer cell line [21], we here find that its paralog, *RPL26L1*, is specifically up-regulated in breast carcinoma. This suggests the possibility that RPL26L1 competes with RPL26 and counteracts its effect on *p53* mRNA translation, thereby providing a permissive environment for cellular transformation. A similar behavior has been observed in zebrafish embryos, where the RP paralogs Rpl22 and Rpl22l1 exert antagonistic effects on *smad1* mRNA translation, thereby having divergent functions in hematopoietic development [57]. In summary, we identified multiple “signatures” of dysregulated RPs in a variety of cancers. These signatures, which seem to originate from copy number variations, include not only over-expressed but also down-regulated RPs and, unexpectedly, have a significant prognostic value.

The control of RP abundances in eukaryotes extends across multiple regulatory layers including transcription [58], direct and indirect splicing-dependent mechanisms [59, 60], translation [61, 62], as well as protein turnover [63]. We have inferred the context-specific expression of RP genes mostly from measurements of mRNA abundance that are more readily available. The need to validate the propagation of these expression patterns to protein level remains, and one hopes it will be addressed by future proteomic studies. It is also important to recognize that other post-transcriptional regulatory layers may contribute to context-specific expression of RPs. For example, RP-encoding mRNAs contain a remarkably diverse set of translation-regulatory signals [64] that are capable of tuning protein abundances. These include specific codon and amino acid usage of individual RPs [65] which are known to influence translation rates both in prokaryotes [66] and eukaryotes [67].

The striking patterns of ribosomal protein expression across cellular contexts reported here highlight the role of individual RPs. As new high-throughput datasets become available and the molecular mechanisms of specific RPs start to be revealed, our understanding of RP-mediated functional specialization becomes clear. So far at least three possible distinct mechanisms seem to be involved: (1) global change in protein synthesis rate [68, 69]; (2) modulation of translation rates of specific mRNAs by ribosomal proteins, independently or as part of the ribosomal complex [17, 21, 29, 70, 71]; and (3) other extra-ribosomal functions of specific ribosomal proteins (reviewed in [7]). Nevertheless, predicting the phenotypes of perturbed RP expression remains very challenging, which suggests that an exciting branch of cellular biology still remains to be discovered.

## Conclusions

Our study reveals an unanticipated plasticity of RP expression across normal and malignant human cells. We found that RPs can help in the identification of cell types and that cancer cells exhibit complex, yet reproducible patterns of RP expression, which could serve as prognostic or diagnostic markers. As evidence for cell type specificity of expression of individual RPs is accumulating, our study provides a foundation for characterizing cellular behaviors that are orchestrated by specific RPs. Additionally, because the regulatory signals that tune RP abundances in individual cell types are not well defined, we anticipate that our study will provide entry points into such studies in the coming years. Ultimately, we hope that a deeper understanding of regulatory mechanisms that are dependent on specific RPs will open up new therapeutic opportunities.

## Methods

### Gene expression datasets

Promoter expression data (normalized relative log expression) for human tissues, primary cells, and cell lines were obtained from the FANTOM5 project [72]. Promoter expression levels were aggregated to yield gene-level expression estimates. To further confirm the consistency of tissue-specific RP gene expression, we used an additional gene expression dataset from the Human Protein Atlas [73].

Gene expression estimates for matched normal and malignant tissue samples were retrieved from The Cancer Genome Atlas (RNASeqV2 RSEM, normalized expression). mRNA expression levels for different cancer cell lines were retrieved from the Cancer Cell Line Encyclopedia (RMA, normalized expression) [74].

RP gene expression data for five different vertebrates (four mammals and one bird) were obtained from [75].

All gene expression datasets were log2-transformed after the addition of a pseudo-count.

### Protein expression dataset

Protein abundance data were retrieved from the Human Protein Atlas [73], which provides qualitative antibody-based measurements of protein expression across 83 different cell types belonging to 44 tissues. Briefly, for each gene, tissue microarray (TMA) immunohistochemical staining was performed using single as well as multiple antibodies for the same protein so as to derive accurate estimates of protein expression. The stained TMA slides were then scored (not detected, low, medium, or high expression) with respect to the intensity of immunoreactivity, the fraction of immunostained cells, and cellular localization of immunoreactivity. The protein expression of RPL39L and RPL3L was probed using 2 and 1 antibodies, respectively. When reporting qualitative protein levels in tissues for which multiple cell types were available, the one displaying higher abundance was selected as done in the Human Protein Atlas [73]. For additional confirmation using an independent dataset, we also retrieved from [76] the protein expression levels that were measured by mass spectrometry from a handful of tissues.

### Proliferation index

For each tissue, we summed the expression levels (transcripts per million) of over 300 genes previously reported to define a cellular proliferation signature [77], which we then normalize between 0 and 1 using the minimum and maximum sums registered across tissues. Importantly, there are only two genes in common between this set of genes and RP genes.

### Specificity score

Following the observation that RP expression levels are strongly correlated between pairs of tissues, we calculated the normalized deviation from the fitted linear relation between the RP expression levels in each tissue and the averaged RP expression levels across all tissues (i.e., the standardized residual). We called this the ”specificity score.” These scores reflect the number of standard deviations (SDs) away from the mean deviation across all RPs. To estimate RP specificity scores in the different hematopoietic cell types, we used the same approach but compared the expression level of RPs in a particular cell type with that in hematopoietic stem cells. We considered that an RP has cell-type-/tissue-specific expression if |specificity score| > 2.5 SD, which, given the normal distribution of the standardized residuals, corresponds on average to the region outside the 97.9 percentiles.

### Relative promoter usage across samples

For each RP gene, we first normalized the expression level of each promoter by the total expression level of all promoters in a given sample (tissue or primary cell), and then computed the mean usage of each promoter across all samples.

### Analysis of transcription factor activity in hematopoietic cell types

We used ISMARA [38] to estimate the activity of 495 different transcription factors in the different hematopoietic cell types. In brief, ISMARA models the expression levels of all mRNAs in terms of the TF binding sites present in the respective gene promoters and the activity of all TFs. The explanatory power of any given TF in a sample is then summarized by its activity z-score. Positive and negative z-scores indicate that genes targeted by that TF are up-regulated and down-regulated in the associated cell type, respectively.

### Analysis of transcription regulatory elements in human RP gene promoters

We obtained the regulatory maps for all human promoters from [38]. Briefly, these maps were constructed by evaluating posterior probabilities of motifs representing the sequence specificity of transcription factors, taking into account the pattern of evolutionary conservation in promoter regions. The data were summarized in a matrix whose elements were the scores of the binding sites for each TF motif (columns) in each promoter region (rows). For any given RP gene, we only considered the regulatory motifs predicted in the promoter showing the highest evidence of expression.

### Transcription factor ChIP analysis

Genomic TF binding sites and respective scores were retrieved from the UCSC Genome Browser, track “Txn Factor ChIP.” TF binding sites were assigned to RP gene promoters if they were localized within 5 kb of the respective transcript start site.

### Dysregulation score

We included in our analysis all cancer types for which matched normal and tumor solid tissue samples were available for at least five patients. For each patient, we computed RP dysregulation scores as the standardized residuals estimated from the linear fit between the RP expression levels in the tumor and normal tissue samples (similar to the specificity score defined above). For each cancer type, we then calculated the median dysregulation score for each RP across all patients. These scores were finally standard score-normalized over all RP genes for each cancer so as to enable their comparison across cancer types. We considered an RP to be dysregulated in a particular cancer if |dysregulation score| > 2.5 SD.

### Copy number variation analysis

Putative copy number alterations estimated using GISTIC 2.0 [78] for all RP genes across different cancers were retrieved from the cBioPortal [79, 80]. Deep and shallow deletions were both classified as deletions, whereas gain and amplifications were grouped as gain alterations. We then calculated the relative frequency of deletion, gain, or no alterations for each RP gene in any given cancer type, and we averaged these numbers across all cancers to yield global estimates of copy number alterations.

### Breast cancer survival analysis

We used the Kaplan-Meier Plotter to calculate the relapse-free survival rates of 3557 patients with breast cancer. Tumor samples were split into “low” and “high” levels with respect to the median gene expression of the RP (or group of RPs) across the cohort.

### Protein sequence alignment

Sequences of ribosomal protein paralogs were aligned using the Needleman-Wunsch global alignment algorithm.

### Statistical analysis

Reported correlation coefficients are Pearson product-moment correlation coefficients. Statistical tests comparing distributions were performed using the non-parametric Mann-Whitney U test.

### Hierarchical clustering

Clustering was performed using Euclidean distance and Ward’s minimum variance method.

### URLs

The following websites are used for reference:

cBioPortal, http://www.cbioportal.org

ENCODE, https://www.encodeproject.org

FANTOM5, http://fantom.gsc.riken.jp/5/data/

ISMARA, https://ismara.unibas.ch/fcgi/mara

Kaplan-Meier Plotter, http://kmplot.com

The Cancer Cell Line Encyclopedia, https://portals.broadinstitute.org/ccle/home

The Human Protein Atlas, http://www.proteinatlas.org

TCGA, http://cancergenome.nih.gov

UCSC Genome Browser, https://genome.ucsc.edu

UniProt, http://www.uniprot.org

## Additional files

Additional file 1: Figures S1-S6. Supplementary figures. (PDF 5509 kb)
Additional file 2: Table S1. Tissue-specific RPs. (XLSX 41 kb)
