# Supplementary material for: Patterns of ribosomal protein expression specify normal and malignant human cells
Source: Genome Biol. 2016 Nov 24;17:236. doi: 10.1186/s13059-016-1104-z (PMC5123215; doi:10.1186/s13059-016-1104-z)
Supplement: Additional file 1: Figures S1-S6. — Supplementary figures. (PDF 5509 kb) [file 13059_2016_1104_MOESM1_ESM.pdf]

Additional file 1: Supplementary figures S1-S6

Patterns of ribosomal protein expression specify normal and malignant human cells

Joao C. Guimaraes[1] and Mihaela Zavolan[1]

[1] Computational and Systems Biology, Biozentrum, University of Basel, 4056 Basel, Switzerland

Correspondence should be addressed to:
J.C.G. (joaoguima@gmail.com) or M.Z. (mihaela.zavolan@unibas.ch)

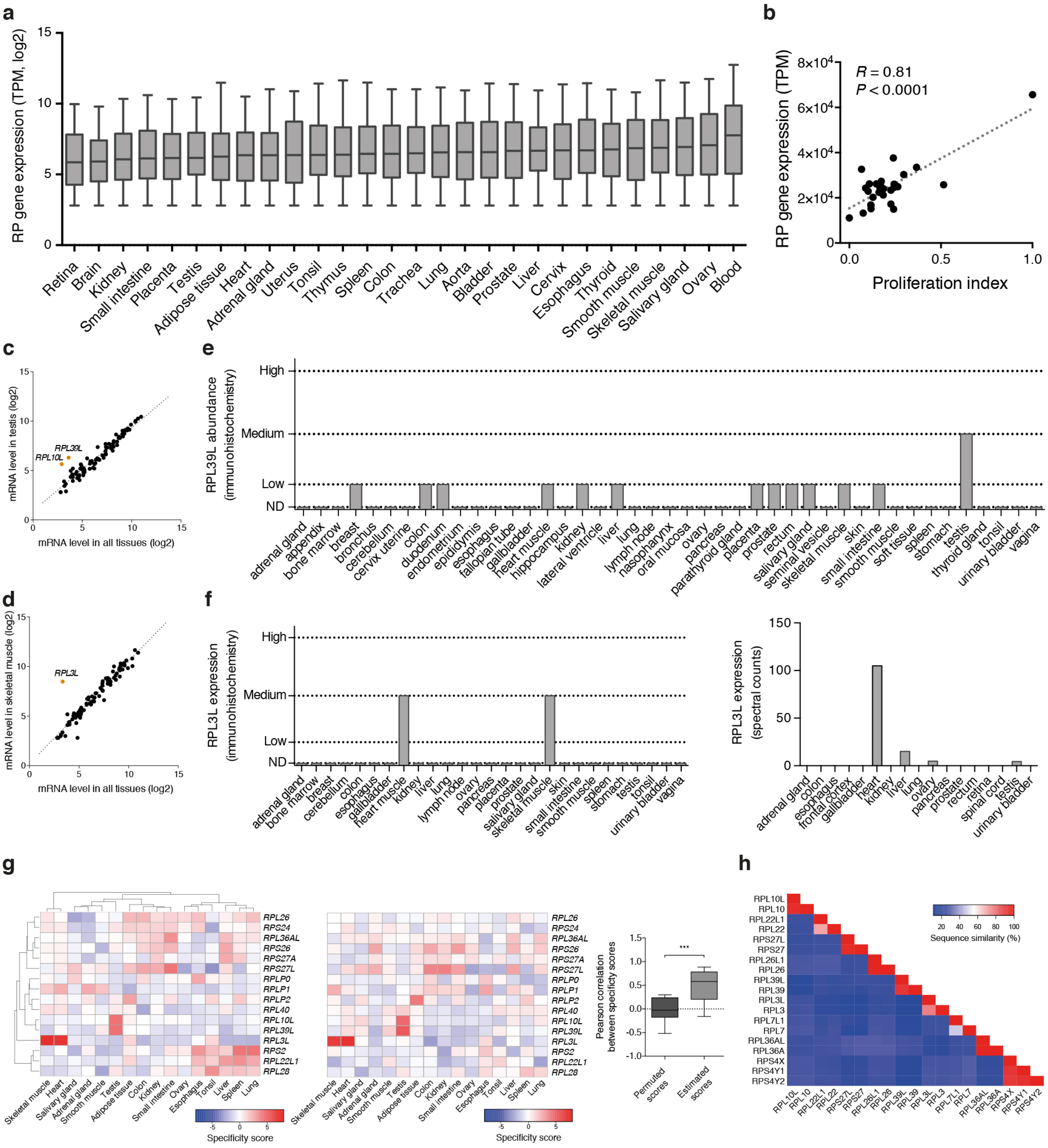

Fig. S1

RP expression specificity across human tissues.

(**a**) Boxplots depicting the distribution of RP mRNA expression levels across human tissues. Boxes extend from the 25th to 75th percentiles (inter-quartile range, IQR), horizontal lines represent the median, whiskers indicate the lowest and highest datum within 1.5*IQR from the lower and upper quartiles, respectively. Tissues have been sorted by their median expression of RP genes. TPM, transcripts per million. (**b**) Total RP gene expression is strongly correlated with the proliferation index of the tissue. (**c**,**d**) RP mRNA expression levels in testis (**c**) and skeletal muscle (**d**) compared to the average expression levels across all tissues. Each dot is an RP gene and the linear fit is shown as a dotted line. RPs displaying significant tissue-specific expression are shown in orange and labeled. (**e**,**f**) The RPL39L protein abundance (**e**) quantified by immunohistochemistry, and the RPL3L protein abundance (**f**) quantified by immunohistochemistry (left) and mass spectrometry (right), show that the tissue specificity of expression that was inferred from mRNA expression measurements is reflected at the protein level. ND, not detected. (**g**) Heat maps of estimated

RP specificity scores using two different datasets: FANTOM5 (left) and The Human Protein Atlas (right). Pearson correlation coefficients between the estimated specificity scores across tissues inferred from the two different datasets for each RPs are significantly higher than those expected by chance. Boxes extend from the 25th to 75th percentiles, horizontal lines represent the median, whiskers indicate the lowest and highest datum within 1.5*IQR from the lower and upper quartiles, respectively. Statistical test comparing the two distributions was performed using the non-parametric Mann-Whitney U test. *** $P < 0.001$ (two-tailed). (**h**) Heat map depicting sequence similarity between the different RP paralogs.

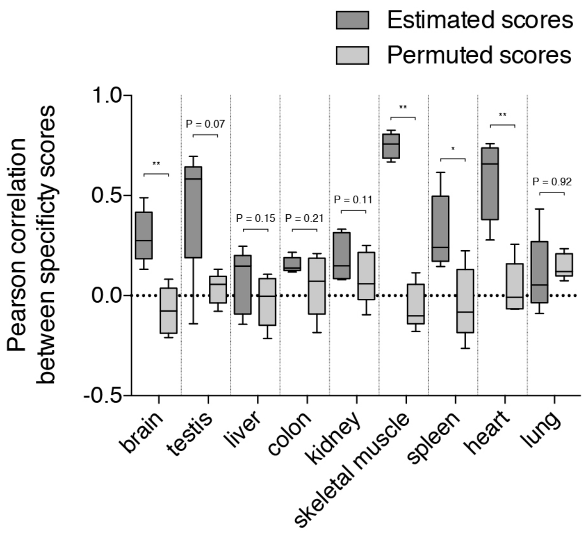

Fig. S2

RPs specificity scores are conserved across species.

Boxplots depict the distribution of Pearson correlation coefficients between the human RP specificity scores and the RP specificity scores in different vertebrates (dark gray). Background distributions of correlation coefficients, computed by permuting the specificity scores of human RPs are shown for comparison (light gray). Boxes extend from the 25th to 75th percentiles, horizontal lines represent the median, whiskers indicate the lowest and highest datum within 1.5*IQR from the lower and upper quartiles, respectively. Statistical test comparing the two distributions was performed using the non-parametric Mann-Whitney U test. * P < 0.05, ** P < 0.01 (one-tailed).

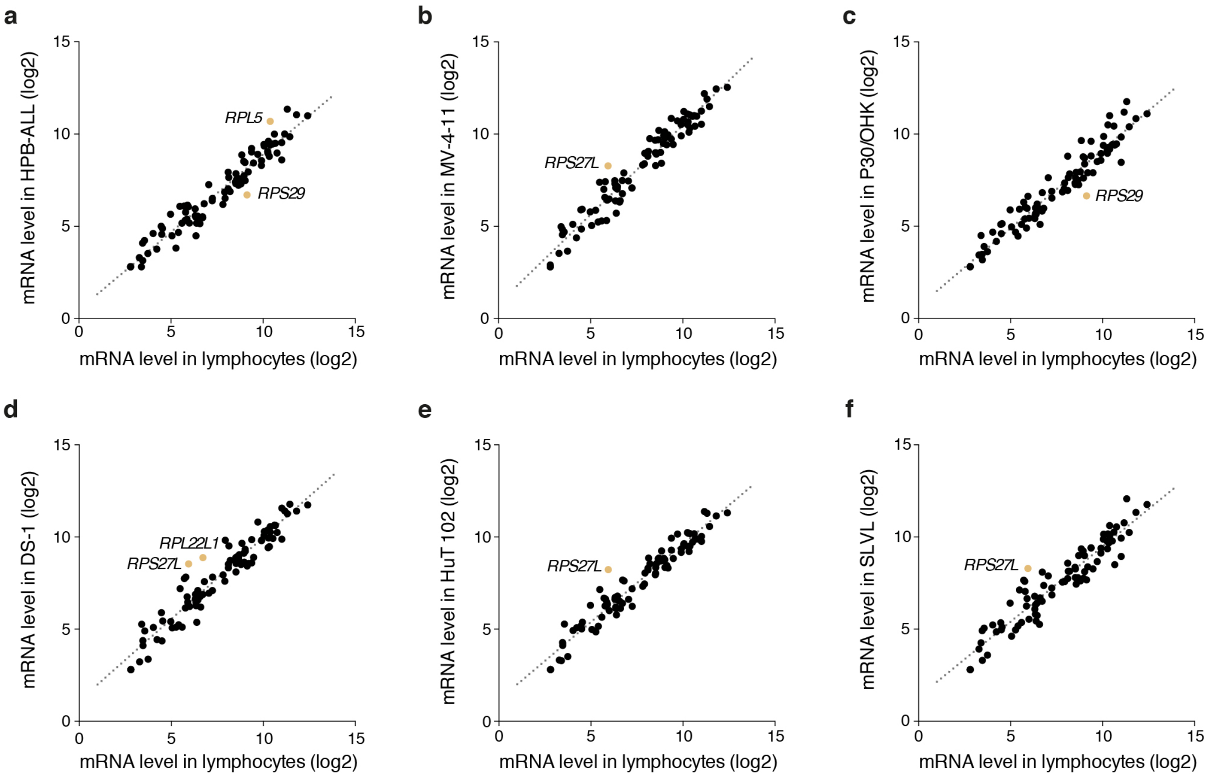

Fig. S3

Dysregulated RP expression in hematopoietic cell lines.

(**a-f**) RP expression levels in different cell line models of lymphoid leukemia (HPB-ALL (**a**), MV-4-11 (**b**) and P30/OHK (**c**)) or lymphoma (DS-1 (**d**), HuT 102 (**e**) and SLVL (**f**)) compared to normal lymphocytes. Each dot is an RP gene and the linear fit is shown as a dotted line. RPs displaying significant dysregulation (i.e. standardized residuals greater than 2.5 s.d.) are shown in orange and have been labeled accordingly.

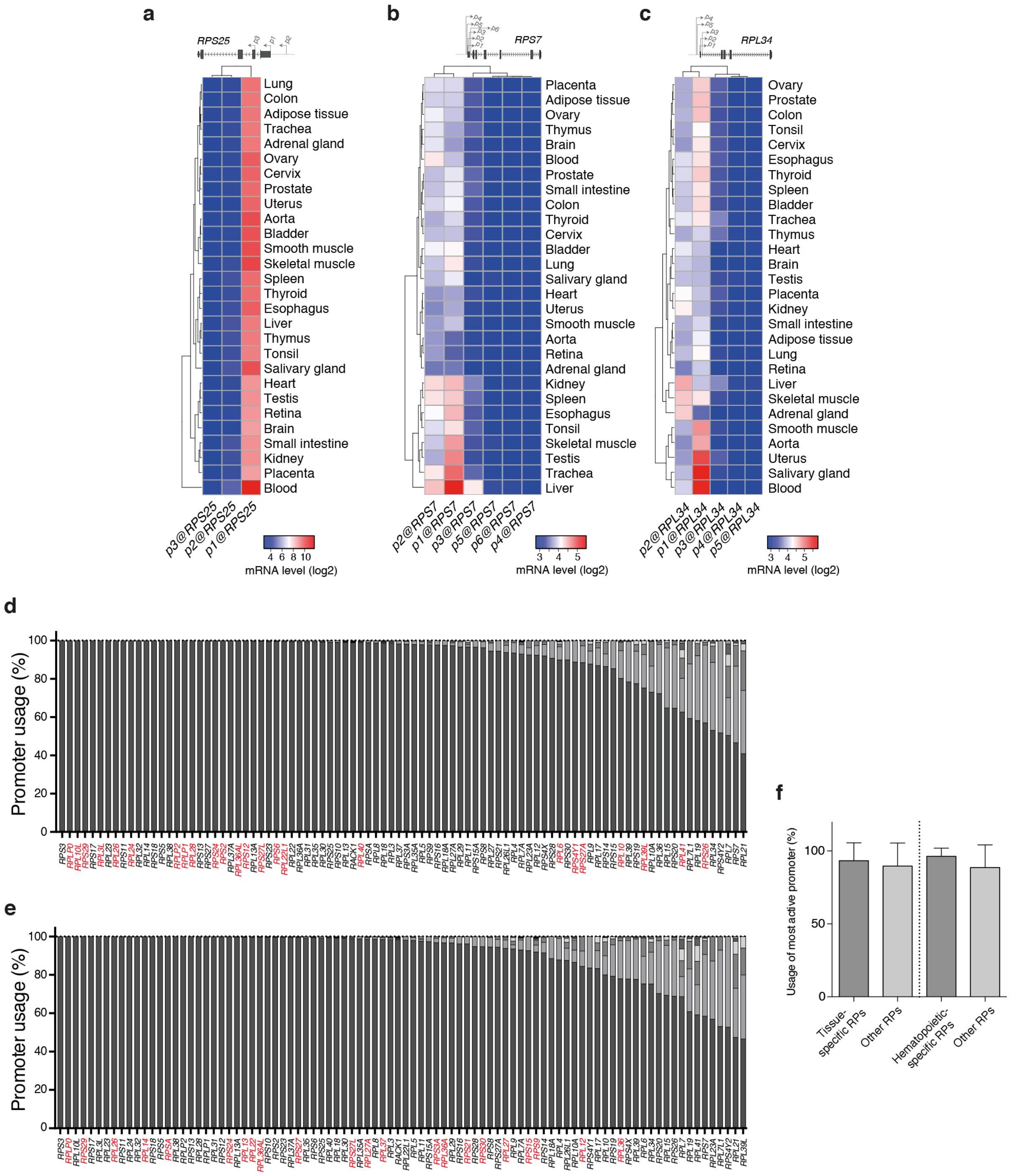

Fig. S4

RP expression specificity is not dictated by the use of alternative promoters.

(**a-c**) Heat maps showing the expression from distinct promoters of three RP genes across tissues. *RPS25* illustrates the typical case of an RP gene with only one active promoter across all tissues (**a**). Correlated use of multiple promoters (*RPS7*) (**b**) and almost mutually exclusive use of alternative promoters (*RPL34*) (**c**) also occur. (**d,e**) Average relative usage of promoters of individual RP genes across different human tissues (**d**) or hematopoietic cells (**e**). Individual promoters are indicated by distinct shades of gray and RPs with evidence of tissue-/cell-type-specific expression are highlighted in red. (**f**) Mean relative usage of the most active promoter of each RP gene across samples shows that RPs with specific expression in individual tissues or hematopoietic samples use predominantly one

promoter, just as RPs without expression specificity. Error bars represent standard deviation across the multiple RPs.

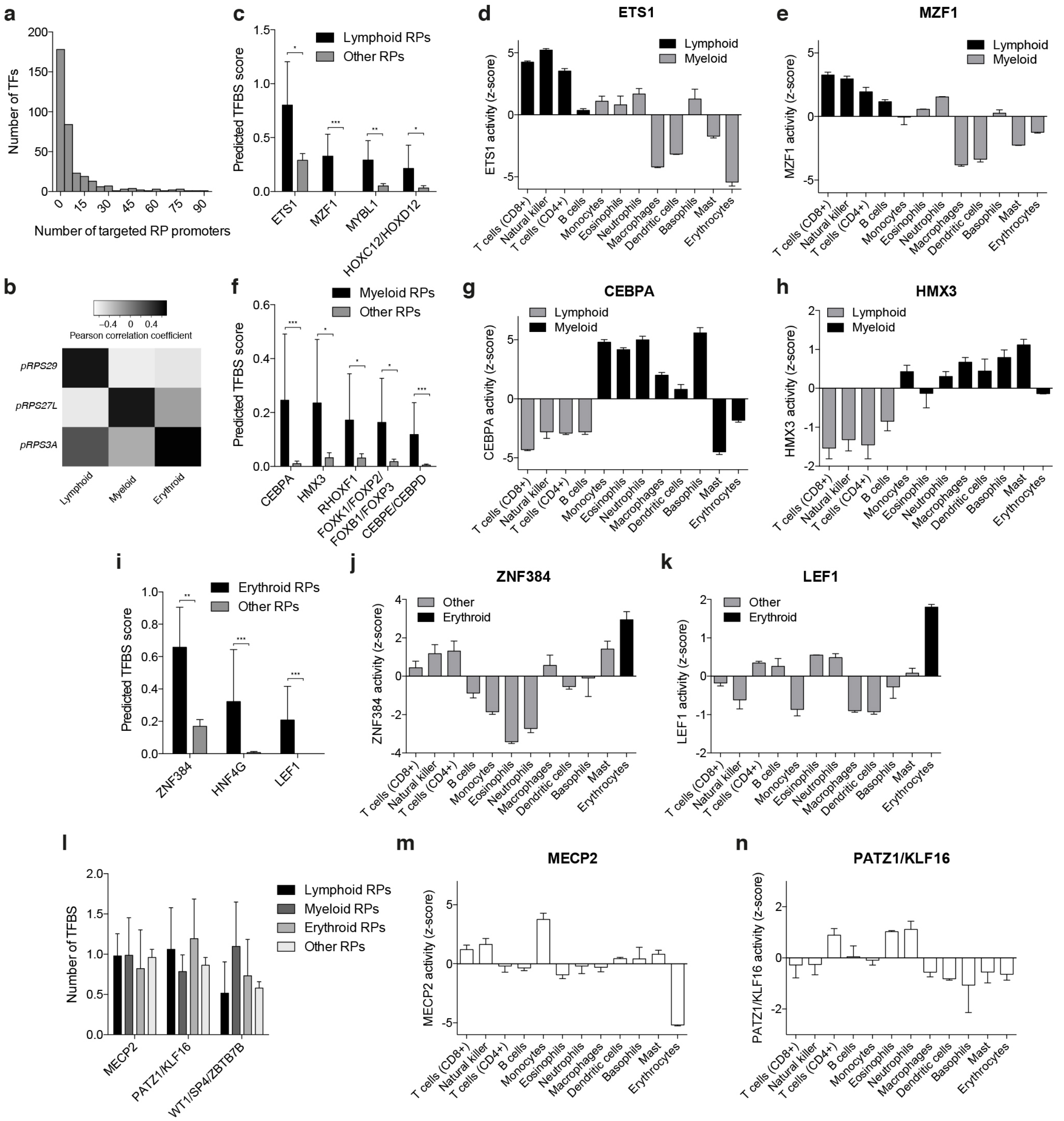

Fig. S5

Regulation of RPs by hematopoietic lineage-specific transcription factors.

(**a**) Histogram of the number of RP promoters targeted by individual TFs shows that most transcriptional regulators bind to just a few promoters. (**b**) Heatmap depicting the Pearson correlation coefficients between the inferred activities of transcription factors in specific lineages (from Fig. 5h) (columns) and their binding scores for promoters of individual RP genes (from Fig. 5g) (rows). (**c**) Average number of transcription factor binding sites (TFBS) predicted in the promoters of different subsets of RPs for four different TFs showing lymphoid-specific activity. Error bars represent the standard error of the mean. Lymphoid lineage-specific RPs: *RPS29*, *RPS27*, *RPL12*, *RPL37* and *RPL26*. (**d, e**) Activity z-scores of ETS1 (**d**) and MZF1 (**e**) in the different hematopoietic cell types. (**f**) Average number of TFBS predicted in the promoter of different subsets of RPs for five different TFs showing myeloid lineage-specific activity. Error bars represent the standard error of the mean. Myeloid lineage-specific RPs: *RPS30*, *RPS24*, *RPS15* and *RPS27L*. (**g, h**) Activity z-scores of CEBPA (**g**) and HMX3 (**h**) in the different hematopoietic cell types. (**i**) Average number of TFBS predicted in the promoter of different subsets of RPs for three different TFs showing erythroid-specific activity. Error bars represent the standard error of the mean. Erythroid

lineage-specific RPs: *RPSA*, *RPL36A*, *RPL27A* and *RPS3A*. (**j, k**) Activity z-scores of ZNF384 (**j**) and LEF1 (**k**) in the different hematopoietic cell types. (**l**) Average number of TFBS predicted in the promoter of different subsets of RPs for three representative TFs that do not show lineage-specific activity. Error bars represent the standard error of the mean. (**m, n**) Activity z-scores of MECP2 (**m**) and PATZ1/KLF16 (**n**) in the different hematopoietic cell types. Statistical test comparing the two distributions of TFBS was performed using the non-parametric Mann-Whitney U test. * $P < 0.05$, ** $P < 0.01$, *** $P < 0.001$ (one-tailed).

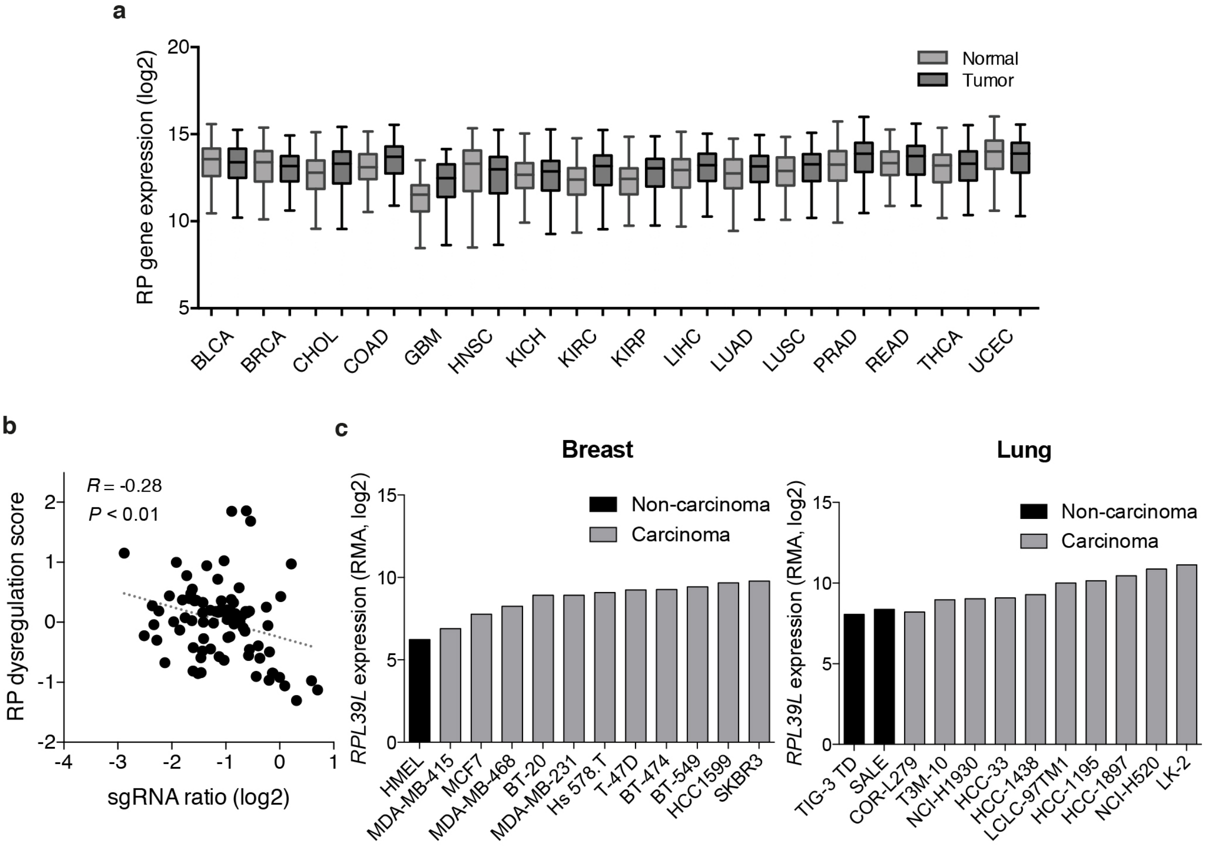

Fig. S6

Expression of RP genes in normal and malignant cells.

(**a**) Boxplots showing the distribution of averaged RP expression levels in the tumor samples (dark gray) and matched normal tissue (light gray) from patients with various types of cancers. Boxes extend from the 25th to 75th percentiles (IQR), horizontal lines represent the median, whiskers indicate the lowest and highest datum within 1.5*IQR from the lower and upper quartiles, respectively. The median expression in tumor samples is almost always higher than the median expression in the corresponding normal tissue samples. The median increase is ~30%. BLCA, bladder urothelial carcinoma; BRCA, breast invasive carcinoma; CHOL, cholangiocarcinoma; COAD, colon adenocarcinoma; GBM, glioblastoma multiforme; HNSC, head and neck squamous cell carcinoma; KICH, kidney chromophobe; KIRC, kidney renal clear cell carcinoma; KIRP, kidney renal papillary cell carcinoma; LIHC, liver hepatocellular carcinoma; LUAD, lung adenocarcinoma; LUSC, lung squamous cell carcinoma; PRAD, prostate adenocarcinoma; READ, rectum adenocarcinoma; THCA, thyroid carcinoma; UCEC, uterine corpus endometrial carcinoma. (**b**) Correlation between the average RP dysregulation score across cancers and the enrichment ratio of the sgRNAs targeting the respective RPs in a CRISPR screen for a melanoma cell line viability. SgRNAs that were positively selected in the screen (i.e. high sgRNA ratio) targeted RPs that are preferentially down-regulated in cancer, whereas sgRNAs that were depleted (i.e. low sgRNA ratio) are associated with RPs that are more often up-regulated in cancer. (**c**) Comparison between *RPL39L* expression level in non-carcinoma (black) and carcinoma (gray) cell line models for breast (left) and lung (right). RMA, Robust Multi-array Average.
